# Association of Preterm Birth and Low Birth Weight With Romantic Partnership, Sexual Intercourse, and Parenthood in Adulthood: A Systematic Review and Meta-analysis

**DOI:** 10.1001/jamanetworkopen.2019.6961

**Published:** 2019-07-12

**Authors:** Marina Mendonça, Ayten Bilgin, Dieter Wolke

**Affiliations:** 1 Department of Psychology, University of Warwick, Coventry, United Kingdom; 2 Psychologische Hochschule Berlin, Berlin, Germany; 3 Division of Mental Health and Wellbeing, Warwick Medical School, University of Warwick, Coventry, United Kingdom

## Abstract

**Importance:**

Social relationships are important determinants of well-being, health, and quality of life. There are conflicting findings regarding the association between preterm birth or low birth weight and experiences of social relationships in adulthood.

**Objective:**

To systematically investigate the association between preterm birth or low birth weight and social outcomes in adulthood.

**Data Sources:**

PubMed, PsycINFO, Web of Science, and Embase were searched for peer-reviewed articles published through August 5, 2018.

**Study Selection:**

Prospective longitudinal and registry studies reporting on selected social outcomes in adults who were born preterm or with low birth weight (mean sample age ≥18 years) compared with control individuals born at term.

**Data Extraction and Synthesis:**

The meta-analysis followed Preferred Reporting Items for Systematic Reviews and Meta-analyses (PRISMA) guidelines. The data were collected and extracted by 2 independent reviewers. Pooled analyses were based on odds ratios (ORs) with 95% confidence intervals and Hedges *g*, which were meta-analyzed using random-effects models.

**Main Outcomes and Measures:**

Ever being in a romantic partnership, ever having experienced sexual intercourse, parenthood, quality of romantic relationship, and peer social support.

**Results:**

Twenty-one studies were included of the 1829 articles screened. Summary data describing a maximum of 4 423 798 adult participants (179 724 preterm or low birth weight) were analyzed. Adults born preterm or with low birth weight were less likely to have ever experienced a romantic partnership (OR, 0.72; 95% CI, 0.64-0.81), to have had sexual intercourse (OR, 0.43; 95% CI, 0.31-0.61), or to have become parents (OR, 0.77; 95% CI, 0.65-0.91) than adults born full-term. A dose-response association according to degree of prematurity was found for romantic partnership and parenthood. Overall, effect sizes did not differ with age and sex. When adults born preterm or with low birth weight were in a romantic partnership or had friends, the quality of these relationships was not poorer compared with adults born full-term.

**Conclusions and Relevance:**

These findings suggest that adults born preterm or with low birth weight are less likely to experience a romantic partnership, sexual intercourse, or to become parents. However, preterm birth or low birth weight does not seem to impair the quality of relationships with partners and friends. Lack of sexual or partner relationships might increase the risk of decreased well-being and poorer physical and mental health.

## Key Points

**Question:**

Are adults who were born preterm or with low birth weight less likely to experience social transitions normative of adulthood, such as romantic partnerships, sexual intercourse, or parenthood?

**Findings:**

In this systematic review and meta-analysis of 21 studies describing up to 4.4 million participants, adults who were born preterm or with low birth weight were less likely to experience a romantic partnership, sexual intercourse, or parenthood than their peers who were born full-term. The likelihood of experiencing these social transitions decreased with lower gestational age and birth weight, and was similar in both young and middle adulthood.

**Meaning:**

The findings suggest that adults who were born preterm or with low birth weight are less likely to have sexual or partner relationships than adults born full-term, which might put them at increased risk of decreased well-being and poorer physical and mental health.

## Teaser

This systematic review and meta-analysis investigates the association between preterm birth or low birth weight and social relationships in adulthood.

## Introduction

Preterm birth or low birth weight (PT/LBW) is associated with an increased risk for disability,^1,2^ neurocognitive impairment,^3,4,5,6^ learning difficulties,^3,6^ and mental health problems,^7,8,9^ with the association being stronger for those with lower gestational age.^3,10,11,12^ These functional deficits are associated with adverse impacts on preterm-born adults’ socioeconomic outcomes.^13^ However, little is known about whether those born preterm master social transitions into adulthood, such as building a supportive peer group, establishing romantic partnerships, having sexual intercourse, or becoming a parent.

Close, intimate, and supportive relationships are associated with increased happiness and well-being,^14,15^ good physical health,^16^ and good mental health.^17^ Studies have shown that social relationships are more challenging for children born PT/LBW.^18^ Indeed, prematurity has been associated with a behavioral phenotype^18,19,20^ and personality profile^21,22,23,24^ that includes being timid, socially withdrawn, overcontrolling, and disinclined toward risk-taking or fun seeking. These differences may predispose PT/LBW individuals to face greater difficulties in establishing romantic and peer relationships.

In contrast, research on social outcomes of adults born preterm is not conclusive. While Scandinavian registry studies have found that adults born PT/LBW were less likely to ever be in a registered partnership^11,12,25^ or to be parents,^12,25^ prospective studies have reported conflicting findings across^26,27,28^ and within^2,29^ studies. Regarding the latter, a Canadian cohort study^2,29^ of extremely low-birth-weight infants reported different findings for social outcomes at distinct time points: while no differences were found in rates of marriage or cohabitation and parenthood between the extremely low-birth-weight individuals and those born full term at ages 22 to 26 years,^29^ adults with extremely low birth weight were less likely to be married or cohabitating and to have had children during the fourth decade of life.^2^ Additionally, there is a lack of research that has analyzed the impact of preterm birth on the quality of close relationships, such as with partners^2,30,31^ and friends.^2,30,32,33,34^

Hence, there are inconsistent and scarce findings about the social lives of PT/LBW adults. This systematic review and meta-analysis systematically investigates the association between being born PT/LBW and social outcomes in adulthood, such as ever being in a romantic partnership, ever having had sexual intercourse, parenthood, quality of romantic relationship, and peer social support. Furthermore, we investigate whether there is a dose-response association according to degree of prematurity and whether outcomes are moderated by type of study (ie, cohort or registry), age, or sex.

## Methods

This meta-analysis followed the Preferred Reporting Items for Systematic Reviews and Meta-analyses (PRISMA) reporting guideline^35^ and was registered with PROSPERO International prospective register of systematic reviews (PROSPERO identifier: CRD42017078286).

### Search Strategy

A systematic search for articles published in the electronic databases PubMed, PsycINFO, Web of Science, and Embase was performed from inception through August 5, 2018, for publications in English. The following keywords were used: (preterm* OR “low birth weight”) AND (partner* OR roman* OR marri* OR sexual* OR reprod* OR fertility OR intercourse OR parent* OR social* OR peer OR friend*) AND (adult*).

### Study Selection Criteria

Studies were eligible for review according to the following criteria: (1) the sample included individuals who were born PT (<37 weeks’ gestation) or LBW (<2500 g at birth); (2) term control group; (3) adult participants (ie, mean sample age ≥18 years); (4) measured at least 1 of the following social outcomes in adulthood: romantic partnership (eg, dating, cohabitation, marriage), quality of romantic relationship (eg, satisfaction, intimacy), sexual intercourse (ie, if ever experienced sexual intercourse), parenthood (ie, if any live biological child), or social support (ie, positive and supportive relationships with friends); and (5) the study was published in a peer-reviewed journal. If data from the same sample were published in multiple works for the same social outcome, we retained (1) the study with the longest follow-up interval (ie, oldest age at assessment); and (2) the study with the largest sample size and the broadest concept coverage.

### Data Collection Process

Two of us (M.M. and A.B.) reviewed titles and abstracts of traced articles. The title and abstract screening was followed by the analyses of full texts to check inclusion criteria. Discordances were resolved by discussion among all authors. When reported information was unclear or numerical data were not obtainable, relevant corresponding authors were contacted for clarification.

### Data Extraction

Studies reporting on PT or LBW were grouped into the same category because infants with low birth weight are mostly born preterm.^36^ When information was available, we used 4 different gestational age subgroups: extremely preterm (EPT; <28 weeks or <1000 g), very preterm (VPT; 28-31 weeks or 1000-1500 g), moderate-to-late preterm (MLPT; 32-36 weeks or 1500-2500 g), and full-term (FT; >36 weeks or >2500 g). When studies referred to preterm birth without mentioning gestational weeks, data were included in the MLPT subgroup.

A standardized form was used to extract data from each study that included publication details, country, characteristics of participants (year of birth, sample size, gestational age or birth weight, percentages of men, and age), type of study (ie, cohort or registry), type of social outcome, and outcome data (ie, means and standard deviations or numbers and frequencies)^37,38,39,40,41,42,43,44,45,46,47,48,49,50,51,52,53,54,55,56,57,58,59,60,61,62,63^ (Table 1). The extraction was conducted independently by 2 of us (M.M. and A.B.) and information was cross-checked for consistency. When inconsistencies emerged information was checked in the original study.

**Table 1.** Summary of the Studies Included in the Meta-analysis of Social Outcomes in Adulthood After Preterm Birth/Low Birth Weight

Source	Country	Year of Birth	Participants, No.		Male, No. (%)		Age Outcome, y	Degree of PT/LBW	Registry or Cohort (Name)	Social Outcomes	Measures
			PT	FT	PT	FT					
Båtsvik et al,^57^ 2015	Norway	1982-1985	37	46	19 (51.4)	25 (54.4)	24	EPT	Cohort	Partnership	Marriage or cohabitation
Cooke,^26^ 2004	United Kingdom	1980-1983	79	71	35 (44.3)	30 (42.3)	19-22	PT	Cohort	Partnership; sexual intercourse, parenthood	Ever in relationship
Dalziel et al,^58^ 2007	New Zealand	1969-1974	126	66	66 (52.3)	33 (50)	31	PT	Cohort (Auckland Steroid Trial)	Partnership	Marriage or cohabitation
Darlow et al,^43^ 2013	New Zealand	1986	230	69	104 (45.2)	33 (47.8)	22-23	VLBW	Cohort	Sexual intercourse	NA
D’Onofrio et al,^11^ 2013	Sweden	1973-2008	154 322	3 146 386	85 195 (55.2)	1 618 442 (51.4)	≤38	EPT, VPT, and MLPT	Registry	Partnership; parenthood	Ever partnered
Drukker et al,^59^ 2018	Israel	1982-1997	4005	53 906	1788 (45)	26 825 (49)	NA	VPT and MLPT	Cohort	Parenthood	NA
Hack et al,^60^ 2002	United States	1977-1979	242	233	116 (48)	108 (46)	20	VLBW	Cohort	Sexual intercourse; parenthood	NA
Hallin et al,^32^ 2010	Sweden	1985-1986	51	52	19 (37.3)	23 (42.6)	18	EPT	Cohort	Peer social support	Adaptive functioning for friends (adult self-report)
Hille et al,^23^ 2008	Netherlands	1983	656	418	294 (44.8)	220 (52.6)	19	VPT	Cohort (POPS Study and Dutch general population)	Partnership; sexual intercourse	In relationship
Husby et al,^33^ 2016	Norway	1986-1988	35	37	14 (40)	15 (40.5)	23	VLBW	Cohort (University Hospital Trondheim)	Peer social support	Adaptive functioning for friends (adult self-report)
Jaekel, et al,^9^ 2017	Germany	1985-1986	200	197	106 (53)	94 (47.7)	26	VPT or VLBW	Cohort (Bavarian Longitudinal Study)	Partnership; sexual intercourse	In relationship
Kajantie et al,^28^ 2008	Finland	1978-1985	162	188	68 (42)	75 (39.9)	22.3	VLBW	Cohort (Helsinki Study of Very Low Birth Weight Adults)	Partnership; sexual intercourse; parenthood	Ever partnered
Kroll et al,^61^ 2017	United Kingdom	1979-1984	122	89	76 (62)	42 (47)	28-34	VPT	Cohort	Partnership; parenthood	In relationship
Männistö et al,^62^ 2015^a^	Finland	1985-1989	397	356	189 (47.6)	170 (47.8)	23.2	MLPT	Cohort (ESTER)	Partnership; sexual intercourse; parenthood	Ever partnered
Mathiasen et al,^25^ 2009	Denmark	1974-1976	1422	192 233	736 (51.8)	98 240 (51.1)	27-29	VPT	Registry	Partnership; parenthood	In relationship
Moster, et al,^12^ 2008	Norway	1967-1983	39 465	828 227	21 715 (55)	421 568 (50.9)	20-36	EPT, VPT, and MLPT	Registry	Partnership; parenthood	Marriage or cohabitation
Odberg et al,^34^ 2011	Norway	1986-1988	134	135	61 (54)	64 (53)	19	LBW (<2000 g)	Cohort	Partnership; peer social support	In relationship; self-reported quality of the social network
Roberts, et al,^63^ 2013	Australia	1991-1992	194	148	84 (45.2)	60 (43.5)	18	EPT or ELBW	Cohort (Victorian Infant Collaborative Study)	Sexual intercourse	NA
Saigal et al,^2^ 2016^a^	Canada	1977-1982	100	89	39 (39.0)	33 (37.1)	32.3	ELBW	Cohort (McMaster ELBW Cohort)	Partnership; quality of romantic relationship; sexual intercourse; parenthood; peer social support	Marriage or cohabitation; satisfaction with partner; Young Adult Social Support Index
Scharf et al,^30^ 2013	Israel	NA	57	57	NA	NA	26.6	PT	Cohort	Quality of romantic relationship; peer social support	Intimacy in relationship; emotional closeness
Winstanley et al,^31^ 2015	United Kingdom	NA	11 592	51 460	3554 (30.7)	8038 (69.3)	31.4	PT	Cohort	Partnership; parenthood; quality of romantic relationship	Marriage or cohabitation; satisfaction with partner

Abbreviations: ELBW, extremely low birth weight (<1000 g); EPT, extremely preterm (<28 weeks’ gestation), FT, full term; LBW, low birth weight (<2500 g); MLPT, moderate-to-late preterm (32-36 weeks’ gestation); NA, not available; PT, preterm; VLBW, very low birth weight (1000-1500 g); VPT, very preterm (28-31 weeks’ gestation).

^a^ This study reported on an early preterm (<34 weeks’ gestation) subgroup overlapping with MLPT subgroup. We excluded this subsample of less than 34 weeks’ gestation from the analysis.

### Quality Assessment

Study quality was assessed independently by 2 of us (M.M. and A.B.) using the Newcastle-Ottawa Scale^37^ (eTable 1 in the Supplement). Scores could range from 0 to 9. The mean (range) of ratings for study quality was 7.3 (4-9), indicating overall good quality.

### Statistical Analysis

Meta-analysis of the overall comparison between adults born PT/LBW and their FT peers was carried out with Comprehensive Meta-analysis version 2 software (Biostat)^38^ for each social outcome. We used pooled odds ratios (ORs) with 95% confidence intervals for studies presenting dichotomous outcomes (eg, frequencies) and Hedges *g* for studies presenting continuous outcomes (eg, means and standard deviations) with random effects. Heterogeneity among studies was assessed with Cochran *Q* (*P* value), Higgins *I*^2^, and τ^2^. Low heterogeneity was defined as an *I*^2^ value of 0% to 25%, moderate heterogeneity as an *I*^2^ of 25% to 75%, and high heterogeneity as an *I*^2^ of 75% to 100%. To explore heterogeneity, we conducted subgroup analyses (dependent on data availability) for degree of prematurity (ie, EPT, VPT, MLPT), type of study (ie, cohort or registry), age groups (ie, young adulthood [18-25 years] or middle adulthood [≥26 years]), and sex.

Publication bias analysis was assessed through (1) the trim and fill procedure to examine the symmetry of effect sizes plotted by the inverse of the standard error^39^ (ideally, effect sizes should mirror one another on either side of the mean); (2) the Begg-Mazumdar rank correlation test to examine the likelihood of bias in favor of small sample size studies,^40^ in which nonsignificance of correlation indicates no publication bias; and (3) Egger test to examine whether publication bias was related to the direction of study findings.^41^ The intercept value provided by this test shows the level of funnel plot asymmetry from the standard precision.

Because PT and LBW were combined into 1 group, it is essential to prove that the findings of the meta-analysis are not dependent on this decision. Therefore, a sensitivity analysis was undertaken in which we repeated the analysis excluding the studies that reported on LBW only.

## Results

### Study Characteristics

**Figure 1.**
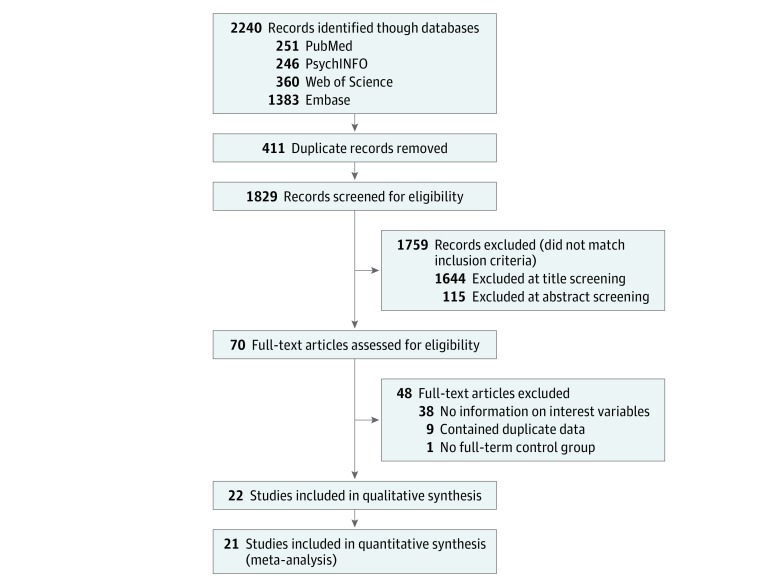
Meta-analysis Flow Diagram One study included in the qualitative synthesis was excluded from meta-analysis because it reported only on number of friends.

Of 1829 articles screened, 21 studies were eligible for quantitative analysis (Figure 1). According to our selection criteria, it was possible to identify 14 studies for romantic partnership, 9 for sexual intercourse, 11 for parenthood, 3 for quality of romantic relationship, and 5 for peer social support. We also identified 5 studies for number of friends,^32,33,34,42,43^ but they were not included in the quantitative synthesis (meta-analysis) owing to the different ways the number of friends was assessed across studies. The studies included in the meta-analysis were conducted in 12 countries (Germany, Denmark, Norway, Sweden, Finland, United Kingdom, Netherlands, Israel, Canada, United States, New Zealand, and Australia). The number of participants included in each analysis of summary data ranged from 4 423 798 (179 724 PT/LBW) for parenthood to 648 (276 PT/LBW) for peer social support (Table 2). Study characteristics are summarized in Table 1. The mean percentage of occurrence of each social transition across the studies is shown in eTable 2 in the Supplement.

**Table 2.** Number of Participants Included in Meta-analysis

Social Outcome	Total No.		No. Analyzed			
	Studies	Participants	Degree of Prematurity	Type of Study	Age Group	Sex
Romantic partnership	14	4 367 489 (176 632 PT)	6244 EPT; 13 606 VPT; 156 782 MLPT	Cohort = 66 566 (13 456 PT); registries = 4 300 923 (163 176 PT)	18-25 y = 2531 (1326 PT); ≥26 y = 356 824 (175 304 PT)	793 Male (435 PT); 967 female (559 PT)
Sexual intercourse	9	3730 (2029 PT)	286 EPT; 1420 VPT; 323 MLPT	NA	18-25 y = 3147 (1732 PT); ≥26 y = 583 (297 PT)	1023 Male (551 PT); 1214 female (685 PT)
Parenthood	11	4 423 798 (179 724 PT)	6207 EPT; 13 369 VPT; 160 148 MLPT	Cohort = 122 952 (16 560 PT); registries = 4 300 917 (163 164 PT)	18-25 y = 1589 (741 PT); ≥26 y = 4 364 369 (174 978 PT)	34 531 Male (2045 PT); 33 101 female (2540 PT)
Quality of romantic relationship	3	63 238 (11 688 PT)	NA	NA	NA	NA
Peer social support	5	648 (276 PT)	NA	NA	NA	NA

Abbreviations: EPT, extremely preterm (<28 weeks’ gestation); MLPT, moderate-to-late preterm (32-36 weeks’ gestation); NA, not analyzed; PT, preterm, VPT, very preterm (28-31 weeks’ gestation).

### Differences in Social Outcomes Between Adults Born PT/LBW and FT

**Figure 2.**
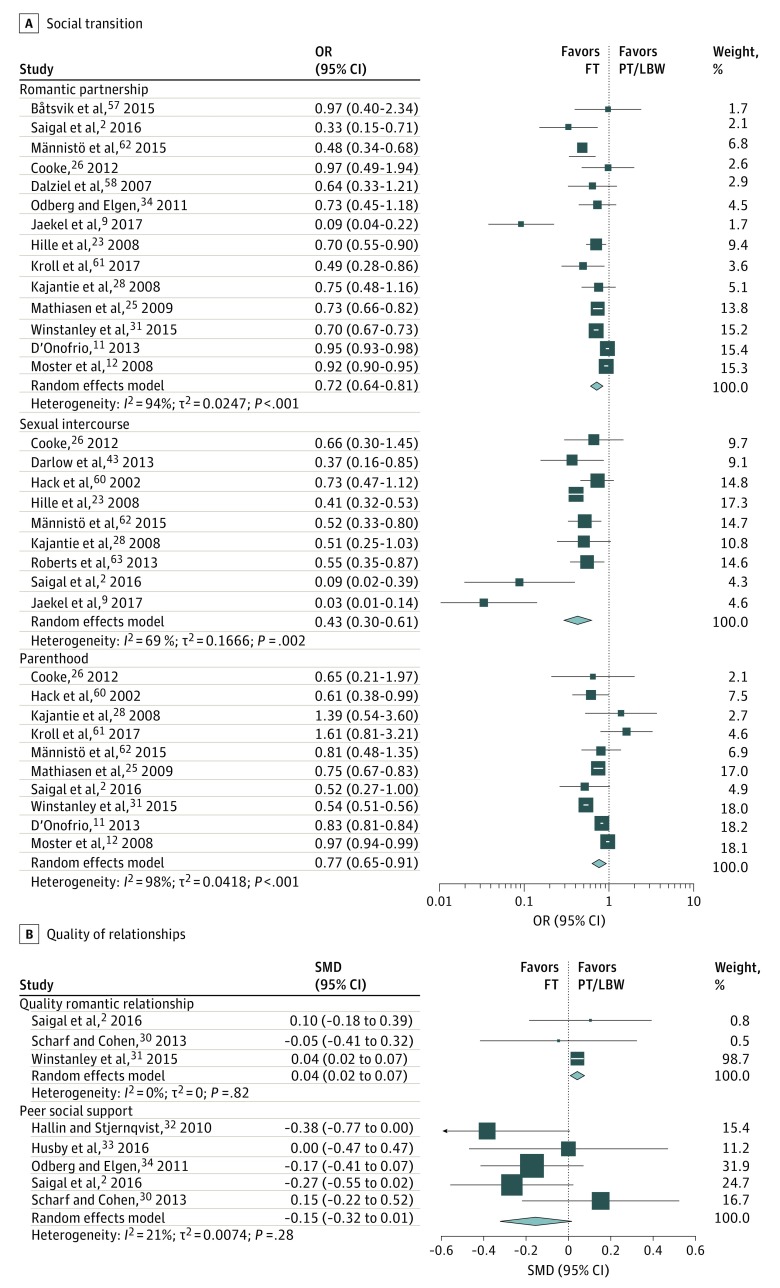
Forest Plots for Social Outcomes of Adults Born Preterm or With Low Birth Weight (PT/LBW) Compared With Adults Born Full-term (FT) Odds ratio (OR) or standardized mean difference (SMD) for individual studies are indicated by squares and 95% CIs by horizontal lines. Pooled estimates and their 95% CIs are represented by diamonds. The size of the squares and the diamonds are proportional to the weight assigned to the relative effect sizes. The arrow for the study of Hallin and Stjernqvist^32^ indicates that the 95% CI exceeds the limit for the effect size range.

Meta-analysis results (Table 3 and Figure 2) revealed that PT adults were less likely to have ever been involved in a romantic partnership than those born FT (OR, 0.72; 95% CI, 0.64-0.81). Heterogeneity analysis indicated high variation in effects between studies. Subgroup analysis according to the degree of prematurity revealed a dose-response association of degree of prematurity and romantic partnership (*Q* = 26.35; *P* < .001) with the EPT subgroup being the least likely to have ever been in a romantic partnership (OR for EPT, 0.33; 95% CI, 0.24-0.50; OR for VPT, 0.67; 95% CI, 0.55-0.82; and OR for MLPT, 0.79; 95% CI, 0.65-0.96). Comparisons of type of study (Table 3) indicated that in both cohort and registry studies PT birth was associated with decreased likelihood of romantic partnership when compared with individuals born FT, but this effect was stronger in cohort studies. In both age groups, PT/LBW were less likely to experience a romantic partnership. Finally, subgroup analysis for sex revealed that both men and women born PT/LBW were less likely to ever be involved in a romantic partnership than their same-sex FT counterpart.

**Table 3.** Associations Between Preterm or Low Birth Weight and Social Outcomes

Social Outcome	Data Points, No.	Hedges *g* or OR (95% CI)	Cochran *Q*	Test for Heterogeneity, *P* Value	τ^2^	*I*^2^*,* % (95% CI)
**Romantic Partnership**						
All studies	14	0.72 (0.57-0.77)^a^	234.39	<.001	0.02	94.45 (92.2-96.05)
Degree of prematurity^b^						
MLPT (32-36 wk GA)	7	0.79 (0.65-0.96)^a^	256.61	<.001	0.03	97.62 (96.58-98.40)
VPT (28-31 wk GA)	7	0.64 (0.48-0.77)^a^	31.25	<.001	0.04	80.80 (61.2-90.51)
EPT (<28 wk GA)	4	0.33 (0.24-0.50)^a^	23.76	<.001	0.39	87.38 (69.84-94.71)
Study type						
Cohort	11	0.65 (0.57-0.73)^a^	20.13	<.001	0.07	68.77 (41.53-83.29)
Registry	3	0.88 (0.80-0.97)^a^	24.01	<.001	0.004	91.67 (78.74-96.74)
Age group						
18-25 y	6	0.69 (0.54-0.90)^a^	5.79	.33	0.008	13.61 (0-38.26)
≥26 y	8	0.73 (0.53-0.78)^a^	217.18	<.001	0.02	96.77 (95.32-97.82)
Sex						
Men	4	0.62 (0.45-0.86)^a^	4.38	.22	0.14	31.52 (0-75.42)
Women	4	0.71 (0.53-0.95)^a^	2.25	.52	0	0 (0-95)
**Sexual Intercourse**						
All studies	9	0.43 (0.31-0.61)^a^	24.81	.002	0.16	69 (35.10-83.9)
Degree of prematurity						
MLPT (32-36 wk GA)	2	0.55 (0.25-1.33)^a^	0.28	.60	0	0
VPT (<32 wk GA)	5	0.37 (0.22-0.66)^a^	18.32	.01	0.28	78.16 (47.61-90.9)
EPT	2	0.32 (0.13-0.87)^a^	5.37	.02	1.37	81.41 (20.87-95.62)
Age group						
18-25 y	7	0.50 (0.42-0.59)^a^	6.35	.38	0.003	5.54 (0-72.4)
≥26 y	2	0.05 (0.02-0.15)^a^	0.86	.35	0	0
Sex						
Men	5	0.49 (0.32-0.78)^a^	9.98	.04	0.22	59.98 (0-85.01)
Women	5	0.45 (0.29-0.69)^a^	3.71	.45	0	0 (0-65.23)
**Parenthood**						
All studies	11	0.78 (0.67-0.90)^a^	555.98	<.001	0.04	98.20 (97.64-98.63)
Degree of prematurity^c^						
MLPT (32-36 wk GA)	5	0.79 (0.65-0.96)^a^	562.88	<.001	0.05	99.11 (98.8-99.34)
VPT (28-31 wk GA)	6	0.67 (0.55-0.82)^a^	65.83	<.001	0.07	90.11 (83.79-94.87)
EPT (<28 wk GA)	3	0.31 (0.23-0.42)^a^	55.26	<.001	0.57	96.38 (92.41-97.46)
Study type						
Cohort	8	0.71 (0.60-0.85)^a^	182.58	<.001	0.09	96.16 (94.21-97.46)
Registry	3	0.85 (0.71-1.01)^a^	131.92	<.001	0.01	98.49 (97.33-99.14)
Age group						
18-25 y	4	0.76 (0.55-1.31)^a^	2.43	.48	0	0 (0-98)
≥26 y	6	0.76 (0.64-0.93)^a^	552.86	<.001	0.12	99.06 (98.77-99.33)
Sex						
Men	5	0.63 (0.36-1.09)^a^	1.68	.79	1.02	0 (0-23.21)
Women	5	0.65 (0.41-1.04)^a^	10.88	.02	0	63.07 (0-88.14)
**Quality of Romantic Relationship**						
All studies	3	0.04 (0.02-0.07)^c^	0.41	.81	0	0 (0-83.64)
**Peer Social Support**						
All studies	5	−0.15 (−0.32 to 0.01)^c^	5.10	.28	0.008	21.63 (0-74.70)

Abbreviations: EPT, extremely preterm; GA, gestational age; MLPT, moderate-to-late preterm; OR, odds ratio; VPT, very preterm.

^a^ Values are odds ratios.

^b^ The number of data points are higher in the degree of prematurity analysis since some studies reported on more than 1 degree of prematurity.

^c^ Values are Hedges *g*.

Being born PT/LBW was associated with being less likely to ever have experienced sexual intercourse (OR, 0.43; 95% CI, 0.31-0.61) (Table 3). Heterogeneity analysis indicated high variation in sexual activity effects between studies. Subgroup analysis for degree of prematurity revealed that both the EPT and VPT subgroups were less likely to ever have had sexual intercourse than the FT adults; however, adults born MLPT did not differ from FT. In both age groups, PT/LBW were less likely to ever have had sexual intercourse than FT individuals, and this association was stronger for the older age group (OR for 18-25 years, 0.50; 95% CI, 0.42-0.59 and OR for ≥26 years, 0.05; 95% CI, 0.02-0.15). Subgroup analysis for sex revealed that both men and women born PT/LBW were less likely to have experienced sexual intercourse than their same-sex counterparts born FT.

There was also a significant association between PT/LBW and parenthood, with adults born PT/LBW less likely to be parents than FT adults (OR, 0.77; 95% CI, 0.65-0.91). Heterogeneity analysis indicated significant variation in parenthood effects between studies. Subgroup analysis for degree of prematurity revealed a dose-response association of degree of prematurity and parenthood (*Q* = 22.30; *P* < .001), with the EPT subgroup being the least likely to have become a parent (OR for EPT, 0.31; 95% CI, 0.23-0.42; OR for VPT, 0.67; 95% CI, 0.55-0.82; and OR for MLPT, 0.79; 95% CI, 0.65-0.96). When comparing the type of study, PT adults were less often reported to be parents in cohort studies, but not in registry studies. Subgroup analysis for age groups revealed no differences between PT/LBW and FT individuals in the younger age group, but PT/LBW adults in the older age group were less likely to be parents compared with FT adults of the same age. No moderation effect was found for sex.

Significant differences between PT and FT adults were found for the quality of romantic relationship (Table 3 and Figure 2). Adults born PT/LBW perceived the relationship with their partner as significantly more satisfying or intimate than those born FT. Heterogeneity was not significant for this variable. Furthermore, we observed no significant differences between PT/LBW and FT adults regarding the peer social support.

### Publication Bias

Under the random-effects model, the point estimate for the combined studies was 0.70 (95% CI, 0.67 to 0.73) for romantic partnership, 0.04 (95% CI, 0.02 to 0.07) for quality of romantic relationship, 0.54 (95% CI, 0.39 to 0.74) for ever having experienced sexual intercourse, 0.78 (95% CI, 0.66 to 0.93) for parenthood, and 0.15 (95% CI, −0.32 to 0.01) for peer social support. With the use of trim and fill, these values remained unchanged for all relational outcomes, indicating no publication bias. The Begg-Mazumdar rank correlation and Egger test were not statistically significant for all outcomes, indicating no evidence of publication bias.

### Sensitivity Analysis

Results remained the same after excluding studies that reported only birth weight. Hence, PT adults were less likely to be in a partnership (OR, 0.75; 95% CI, 0.66-0.85), to have ever had sexual intercourse (OR, 0.48; 95% CI, 0.31-0.76), and to be parents (OR, 0.80; 95% CI, 0.67-0.97) in comparison with FT adults.

## Discussion

Our findings revealed that adults born PT/LBW are less likely to experience romantic partnerships, sexual intercourse, or parenthood. Nevertheless, when they were in a romantic partnership or had friends, the quality of these relationships was similar to those experienced by FT adults.

Using summary data from prospective studies with more than 4 million participants provided evidence for a temporal association between being born PT/LBW and establishing social transitions into adulthood, here defined as romantic partnership, sexual intercourse, and parenthood. The associations were robust across degree of prematurity, age groups, and sex. These findings are consistent with the increasing recognition of the impact that early life influences have on outcomes in adulthood.^13,44,45^ Furthermore, our findings are in line with evidence of a preterm behavior phenotype that follows into adulthood,^21,22,24^ which might be associated with more difficulty engaging in these transitions for individuals born PT/LBW.

We verified that the strength of the associations between PT/LBW and social transitions were in general small for romantic partnership and parenthood and moderate for sexual intercourse. The associations diverged depending on degree of prematurity, type of study, and age group. The subgroup analysis for degree of prematurity revealed that the likelihood of PT/LBW experiencing a romantic partnership, sexual intercourse, or parenthood decreased with lower gestational age. Indeed, a significant dose-response association was found between degree of prematurity and rates of romantic partnership and parenthood, with adults born EPT 67% less likely to be in a romantic partnership and 69% less likely to be parents than those born FT.

With respect to the type of study, we found that PT/LBW adults were less likely to have experienced romantic partnership or parenthood in cohort studies compared with registry studies. This difference may be related to the fact that cohort studies included mainly individuals born at less than 32 weeks’ gestational age, whereas registry studies included the full range of preterm birth, and the likelihood of occurrence of these transitions decreases with lower gestational ages.

It has been suggested that PT/LBW individuals might take longer to accomplish the milestones normative of adult life, such as employment, romantic partnership, and parenthood.^28^ The current findings do not support this hypothesis. We verified that the difference of experiencing these transitions in comparison with FT individuals did not alter in the older age group and, in some cases, it was even greater in the older age group. To illustrate, while at ages 18 to 25 years, individuals born PT/LBW were 50% less likely than those born FT to have ever experienced sexual intercourse, after the age of 25 years the decreased likelihood for PT/LBW went up to 95%. These findings may be cautiously interpreted, as only 2 studies^2,9^ could be included in the older age group for this analysis. Alternatively, we may speculate that new ways of dating, such as dating applications, may be used more often by the younger age group of PT/LBW individuals.^46^

Adults born PT/LBW were overall less likely than those born FT to be parents. However, this difference was not significant in the younger age group. This finding is in line with the findings of Saigal et al.^2^ A likely explanation is that, consistent with the general trend for first parenthood to take place in the late 20s or early 30s,^47^ few participants in the FT group were parents, yielding no difference between groups. However, once parenthood was assessed in middle adulthood, the differences between PT/LBW and FT groups emerged. At a societal or population level, it suggests that prematurity is associated with a cross-generational fertility loss. Adults born PT/LBW are less likely to become parents and their parents were already less likely to have subsequent children after their preterm child was born.^48^

Overall, rather than a delay, our findings suggest persistent difficulties in making these social transitions that have been associated with negative outcomes later in life,^49,50^ such as lower wealth, social isolation, and poorer physical and mental health. Both biological and environmental factors, such as alterations in the so-called social brain as part of the neurodevelopmental sequelae of preterm birth^51^ or parental stress in the early stages of life,^52^ have been found to contribute to social difficulties for PT/LBW individuals, such as being more timid and withdrawn. However, more investigation is required to shed light on the mechanisms through which biological and environmental factors interplay during PT/LBW individuals’ development. These findings highlight, on the one hand, the need for more prospective studies over the life course of PT/LBW individuals and the analysis of early predictors of social outcomes and, on the other hand, the importance of continued monitoring and adequate support of PT/LBW individuals throughout life.

With respect to sex, it was only possible to include 4 to 5 studies in these subgroup analyses. We verified that both men and women born PT/LBW were less likely to have experienced romantic partnerships or sexual intercourse than their counterparts born FT. No differences were found for parenthood; however, it is important to note that there were few participants with children in this subgroup analysis. Previous studies have not been consistent when analyzing the role of sex on social outcomes.^2,26,28^ Although it was possible to pool data from more than 1200 participants in these analyses, the lack of studies reporting on sex highlight the need for future research to clarify its moderating role.

We found that PT/LBW individuals perceived their romantic relationships slightly more positively than FT individuals, and that there was no difference for perceptions of peer social support between both groups. Although it was not possible to assess the amount of friends in this meta-analysis, most studies have found that PT/LBW adults had fewer friends^42,43,53^ than FT adults. In addition, studies on PT/LBW children and adolescents reported poorer-quality relationships with peers^18,54^ than those born FT, including being bullied by peers more often.^55^ Hence, our findings suggest that despite fewer close relationships, relationship quality was not poorer when PT/LBW adults had friends or a partner, or the quality of relationships in PT/LBW individuals improves into adulthood. Longitudinal studies are required to explore these alternative explanations.

### Limitations

This comprehensive study of social outcomes of premature birth uses large sample sizes of PT/LBW individuals in comparison with FT individuals, particularly in the analysis of romantic partnership and parenthood. However, there are limitations for the other outcome measures—having ever experienced sexual intercourse, quality of romantic relationships, and peer social support—that included a smaller number of studies and PT/LBW participants. There are also considerable variations of how peer support and quality of romantic relationships were measured across studies. For example, quality of romantic relationships included studies reporting on satisfaction with partner and intimacy, and social support included studies reporting on emotional closeness with friends to self-reported quality of social network. We recommend individual studies use similar valid measures to make comparisons less problematic.

Furthermore, the degree of prematurity is associated with physical and mental health and cognitive development,^11,12,13,45^ and information on disability was not available for most studies. Thus, it could not be assessed whether functional deficits or disability moderated the association between PT/LBW and social outcomes. In this study, PT and LBW were treated as 1 factor. Although these constructs show high comorbidity and our sensitivity analyses revealed consistent results, it would be important to disentangle the effects of PT and LBW and their possible additive effects on social outcomes. This would involve considering data on birth weights appropriate for gestational age or small for gestational age, which most studies included in the meta-analyses did not report. Future research should address these limitations by conducting individual participant meta-analysis and obtaining data directly from the study authors.

The heterogeneity of studies was high, indicating considerable variation. This might arise from incorporating cohort and registry studies with various sample sizes. To address this possibility, we used a random-effects model in the analysis and conducted moderator analyses. Nevertheless, our moderator analysis explained only some of the heterogeneity. Thus, the findings from the current study should be interpreted with caution and analysis should be repeated when more adulthood data becomes available from the cohort studies. Also, only English publications were considered in this meta-analysis and, therefore, potential language bias should be taken into account.

## Conclusions

This systematic review and meta-analysis provides a qualitative and quantitative overview of the current state of knowledge concerning social outcomes in adults born PT/LBW. Pooling data from multiple cohort and registry studies provided evidence that fewer adults born PT/LBW experience romantic partnerships, sexual intercourse, or parenthood. These associations are stronger the lower the gestational age and were found in young and middle adulthood. However, when PT/LBW individuals were in a romantic partnership or had friends, the quality of these relationships was at least as good compared with FT individuals. Hence, analyzing both objective indicators about the occurrence of social transitions and subjective measures about the quality of close relationships provided distinct and complementary information on the social lives of adults born PT. The implications of the current findings are that PT/LBW adults are at increased risk of never experiencing sexual intercourse, being without a supportive partner, and being less likely to experience parenthood. Lack of sexual activity^56^ and lack of romantic partner support^9^ are associated with lower levels of happiness and poorer physical and mental health. Future research is needed to identify the predictors and promotive factors of social outcomes in PT/LBW individuals to allow for timely interventions in aiding the transition into adulthood.

## Notes

Supplement. **eTable 1.** Newcastle-Ottawa Quality Assessment Scale—Cohort Studies **eTable 2.** Mean Percentage of Occurrence of Social Transition
