# Supplementary material for: Association of Preterm Birth and Low Birth Weight With Romantic Partnership, Sexual Intercourse, and Parenthood in Adulthood: A Systematic Review and Meta-analysis
Source: JAMA Netw Open. 2019 Jul 12;2(7):e196961. doi: 10.1001/jamanetworkopen.2019.6961 (PMC6628597; doi:10.1001/jamanetworkopen.2019.6961)
Supplement: Supplement. — eTable 1. Newcastle-Ottawa Quality Assessment Scale—Cohort Studies eTable 2. Mean Percentage of Occurrence of Social Transition [file jamanetwopen-2-e196961-s001.pdf]

Supplementary Online Content

Mendonça M, Bilgin A, Wolke D. Association of preterm birth and low birth weight with romantic partnership, sexual intercourse, and parenthood in adulthood: a systematic review and meta-analysis. *JAMA Netw Open*. 2019;2(7):e196961. doi:10.1001/jamanetworkopen.2019.6961

eTable 1. Newcastle-Ottawa Quality Assessment Scale—Cohort Studies
eTable 2. Mean Percentage of Occurrence of Social Transition

This supplementary material has been provided by the authors to give readers additional information about their work.

© 2019 Mendonça M et al. *JAMA Network Open.*

eTable 1. Newcastle-Ottawa Quality Assessment Scale—Cohort Studies

**Selection**				**Comparability**	**Outcome**		
Representativeness of the exposed cohort<br>a) truly representative of the average ______________ (describe) in the community *****<br>b) somewhat representative of the average ______________ in the community *****<br>c) selected group of users eg nurses, volunteers<br>d) no description of the derivation of the cohort	Selection of the non-exposed cohort<br>a) drawn from the same community as the exposed cohort *****<br>b) drawn from a different source<br>c) no description of the derivation of the non-exposed cohort	Ascertainment of exposure<br>a) secure record (eg surgical records) *****<br>b) structured interview *****<br>c) written self-report<br>d) no description	Demonstration that outcome of interest was not present at start of study<br>a) yes *****<br>b) no	Comparability of cohorts on the basis of the design or analysis<br>a) study controls for ______________ (select the most important factor) *****<br>b) study controls for any additional factor ***** (This criteria could be modified to indicate specific control for a second important factor.)	Assessment of outcome<br>a) independent blind assessment *****<br>b) record linkage *****<br>c) self-report<br><br>d) no description	Was follow-up long enough for outcomes to occur<br>a) yes (select an adequate follow up period for outcome of interest) *****<br>b) no	Adequacy of follow up of cohorts<br>a) complete follow up - all subjects accounted for *<br>b) subjects lost to follow up unlikely to introduce bias - small number lost - > ____ % (select an adequate %) follow up, or description provided of those lost) *<br>c) follow up rate < ____% (select an adequate %) and no description of those lost<br>d) no statement

© 2019 Mendonça M et al. *JAMA Network Open.*

eTable 2. Mean Percentage of Occurrence of Social Transition

		Romantic partnership (%)	Sexual Intercourse (%)	Parenthood* (%)
All	PT	37.3	69.2	20.1
	FT	54.7	80.9	22.2
Degree of prematurity	EPT	25.2	55.8	17.3
	VPT	37.5	70.8	21.6
	MLPT	44.1	78.6	20.2
Type of study	Cohort PT	51.6	NA	15.2
	Cohort FT	61.8	NA	18.9
	Registry PT	15.1	NA	24.8
	Registry FT	28.2	NA	35.4
Age Group	18-25 PT	44.4	66.7	9.4
	18-25 FT	53.6	76	10.5
	≥ 26y PT	33.1	77.9	24.5
	≥ 26y FT	56.1	98.4	32.7
Sex participant	Male PT	41.7	68.9	8
	Male FT	48.6	81.9	10.6
	Female PT	51.2	70.8	15
	Female FT	61.2	84.3	18.9

*Percentages for parenthood exclude data from Drukker et al. because it reports on number of offspring rather than if parent.

© 2019 Mendonça M et al. *JAMA Network Open.*
